# Gut Microbiota Diversity and Human Diseases: Should We Reintroduce Key Predators in Our Ecosystem?

**DOI:** 10.3389/fmicb.2016.00455

**Published:** 2016-03-31

**Authors:** Alexis Mosca, Marion Leclerc, Jean P. Hugot

**Affiliations:** ^1^Hôpital Robert Debré, Assistance Publique-Hopitaux de ParisParis, France; ^2^Institut National de la Santé et de la Recherche Médicale et Université Paris Diderot, Sorbonne Paris-Cité, United Medical Resources 1149 Labex InflamexParis, France; ^3^INRA, AgroParisTech, United Medical Resources 1319 MICALISParis, France

**Keywords:** dysbiosis, ecosystem, predator, western lifestyle, chronic human conditions, *Bdellovibrio bacteriovorus*

## Abstract

Most of the Human diseases affecting westernized countries are associated with dysbiosis and loss of microbial diversity in the gut microbiota. The Western way of life, with a wide use of antibiotics and other environmental triggers, may reduce the number of bacterial predators leading to a decrease in microbial diversity of the Human gut. We argue that this phenomenon is similar to the process of ecosystem impoverishment in macro ecology where human activity decreases ecological niches, the size of predator populations, and finally the biodiversity. Such pauperization is fundamental since it reverses the evolution processes, drives life backward into diminished complexity, stability, and adaptability. A simple therapeutic approach could thus be to reintroduce bacterial predators and restore a bacterial diversity of the host microbiota.

## Introduction

The gut is the largest interface (200 m^2^) between the host and its external milieu. It plays an important part in the metabolism of nutriments and water absorption. Furthermore, immune cells of the Gut Associated Lymphoid Tissue (GALT) are more numerous than those from other secondary lymphoid tissues contained in the whole body. They interact with trillions of bacteria, archaea and eukaryotes forming a complex ecosystem: the gut microbiota. Almost 80% of microbes observed by microscopic examination of fecal specimens are not recoverable by culture (Suau et al., 1999). Thus, until the recent development of culture-independent methods, which combine isolation and sequencing of nucleic acids and powerful bioinformatics analyses, the gut microbiota was seen as an ignored organ or a black box.

Our knowledge on the Human gut microbiota is quickly increasing. In a given individual, the microbiota is relatively stable after the first months of life (Koenig et al., 2011). At the population level, the microbiota is also supposed to be stable and selected by evolution (Ochman et al., 2010; Jalanka-Tuovinen et al., 2011). However, the commensal microbiota can be qualitatively and quantitatively modulated by the environment (Huttenhower et al., 2012). Recent changes in the environment may thus have altered the mutually beneficial interaction toward another stable but harmful balance (Lozupone et al., 2012; Shade et al., 2012).

Sometimes and unexpectedly, links have been established between diseases of industrialized countries and altered patterns of the gut microbial ecosystem collectively known as **intestinal dysbiosis**. The concept of dysbiosis refers to an unbalanced microbiota, which is most of the time supposed to be harmful. The effectiveness of fecal microbiota transplantation in the cases of *Clostridium difficile* associated colitis or T2D supports this opinion (Vrieze et al., 2012; Sha et al., 2014). As a result, “understanding the structure and function of the human symbiont communities might become the first great breakthrough of twenty-first century medicine” (Guarner, 2014).

Loss of microbiota **diversity** (LOMD) appears as the most constant finding of intestinal dysbiosis. By analogy with macro ecosystems, we propose here that it is related to the loss of bacterial predators associated with our modern Western lifestyle. Interestingly, this hypothesis suggests that the reintroduction of bacterial predators in our digestive ecosystem may be an option for improving/restoring the gut microbiota diversity and for treating at-risk people (Jakobsson et al., 2014).

## Human diseases, western lifestyle and microbiota diversity: A tripartite association

### Human diseases are often associated with the modern western lifestyle

**Figure 1:**
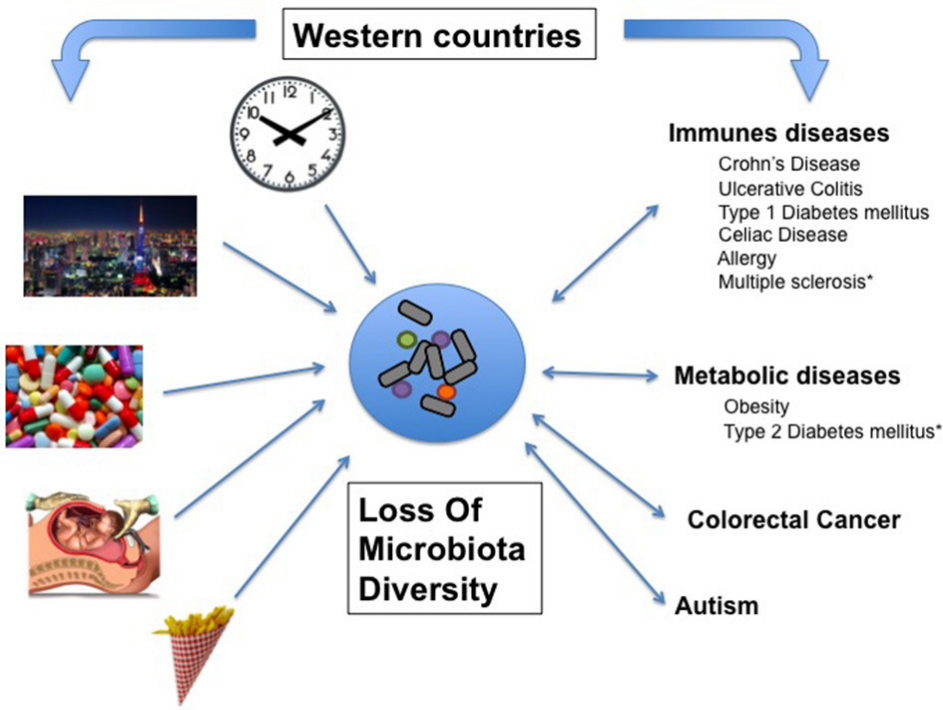
**Associative links between Western lifestyle, Human conditions, and loss of microbial diversity (LOMD)**. On one hand, most of the Human diseases affecting westernized countries are associated with LOMD and on the other hand, some western lifestyle patterns cause LOMD. Then, LOMD appears to play a central role linking western lifestyle and western chronic human conditions (see also Table 1). ^*^LOMD not assessed.

In the past decades, the prevalence of numerous diseases has sharply increased, a phenomenon initially observed in Western countries and more recently in developing countries (Figure 1, Table 1). These complex genetic disorders include “immune related” diseases like allergy, inflammatory bowel diseases (IBD), type 1 diabetes mellitus (T1D), multiple sclerosis (MS) but also colorectal cancer or metabolic disorders like obesity and type 2 diabetes mellitus (T2D; Mayr et al., 2003; Eder et al., 2006; Harjutsalo et al., 2008; Andersson et al., 2014). Unrecognized environmental changes necessarily explain these outbreaks. The higher occurrence of some of these diseases (i.e., asthma, T1D, MS, obesity, T2D, colorectal cancer) among migrants (Bodansky et al., 1992; Hammond et al., 2000; Grüber et al., 2002; Pinheiro et al., 2009; Creatore et al., 2010), especially when emigrating before the age of 5 years (Kuehni et al., 2007) confirms a role of early environmental risk factors.

**Table 1:** **Human diseases and western lifestyle: prevalence and LOMD**.

**Disease**	**LOMD**	**References**	**Increased prevalence in western countries**	**References**
**IMMUNE DISEASES**				
Crohn's Disease	+	Manichanh et al., 2006; Dey et al., 2013; Sha et al., 2013; Matsuoka and Kanai, 2015	+	Lehtinen et al., 2011
Ulcerative Colitis	+	Michail et al., 2012; Sha et al., 2013	+	Lehtinen et al., 2011
Type 1 diabetes mellitus	+	Giongo et al., 2010; De Goffau et al., 2013; Kostic et al., 2015	+	Harjutsalo et al., 2008; Andersson et al., 2014
Multiple Sclerosis	ND	Bhargava and Mowry, 2014	+	Mayr et al., 2003
Celiac Disease	+	Schippa et al., 2010	+	Lohi et al., 2007
Allergy	+	Wang et al., 2008; Ismail et al., 2012; Abrahamsson et al., 2014	+	Latvala et al., 2005; Eder et al., 2006
**METABOLIC DISEASES**				
Obesity	+	Turnbaugh et al., 2009	+	WHO | Obesity and overweight
Type 2 diabetes mellitus	-		+	WHO | Diabetes
**CANCER**				
Colorectal cancer	+	Ahn et al., 2013	+	European Cancer Observatory http://eco.iarc.fr/
**OTHERS**				
Irritable Bowel Syndrome	+	Carroll et al., 2012; Durbán et al., 2012	–	Lovell and Ford, 2012
Recurrent *Clostridium difficile* Infection	+	Chang et al., 2008	ND	
Autism	+	Kang et al., 2013	+	Atladottir et al., 2015
Necrotising Enterocolitis	+	Stewart et al., 2013	ND	
Graft Versus Host Disease	+	Jenq et al., 2012	ND	

*WHO | Diabetes WHO. Available at: http://www.who.int/mediacentre/factsheets/fs312/en/ [Accessed September 29, 2015]*.

*WHO | Obesity and overweight WHO. Available at: http://www.who.int/mediacentre/factsheets/fs311/en/ [Accessed September 29, 2015]*.

*ND, No Data*.

### The reduction in intestinal microbiota diversity is associated with human diseases

Among the huge body of literature describing disease-associated microbiota, LOMD appears as a common feature of most dysbioses. In digestive diseases such as Crohn's disease (CD) (an IBD condition; Sha et al., 2013; Matsuoka and Kanai, 2015), irritable bowel syndrome (IBS), with (Carroll et al., 2012) or without diarrhea (Durbán et al., 2012) and colorectal cancer (Ahn et al., 2013), LOMD is constantly observed. The duodenum-associated microbiota of celiac disease patients is also less diversified (Schippa et al., 2010). LOMD is a risk factor of relapse in *Clostridium difficile* colitis (Chang et al., 2008). Obesity is associated with altered representations of specific bacterial species, which often differ between studies, but LOMD is constantly retrieved with up to 20% loss of phylogenetic diversity and one third difference in terms of gene number (Turnbaugh et al., 2009; Cotillard et al., 2013; Tagliabue and Elli, 2013). Finally and more surprisingly, LOMD is also reported in non-digestive diseases such as autism (Kang et al., 2013). It thus appears that LOMD is a general feature associated with several Human conditions.

However, these associations might be the consequence rather than the cause of the disease. Several authors have questioned this chicken-egg problem and found arguments for a causal effect of LOMD on Human diseases. LOMD was found in CD (54 ribotypes in CD patients vs. 88 in healthy controls) not only in case of flare but also in case of remission (Manichanh et al., 2006) suggesting that it is not a consequence of gut inflammation. In healthy people investigated for T1D markers, the microbial diversity was lower in fecal samples of children with at least two disease-associated autoantibodies when compared to autoantibody-negative children matched for age, sex, early feeding history, and HLA-genotyping (De Goffau et al., 2013). In two studies following children at risk for T1D longitudinally from birth, a decrease of microbial diversity occurred just before the occurrence of anti-islet cell antibodies and subsequently T1D (Giongo et al., 2010; Kostic et al., 2015). Among obese individuals, a decrease of richness assessed by a low microbiome gene count was predictive of response to diet (Cotillard et al., 2013).

Consistently, in two prospective studies, LOMD measured during the first week of life was predictive of allergic manifestations at ages of 12 and 18 months (Wang et al., 2008; Ismail et al., 2012) and asthma at the age of 7 years (Abrahamsson et al., 2014). Decrease of microbial diversity also predicts the response to treatments. For example, in Ulcerative Colitis (UC), children who responded to corticosteroids had a more diverse microbiota than non-responders (Michail et al., 2012). LOMD is also a risk factor of relapse after intestinal resection in CD (Dey et al., 2013). Finally, it seems that CD can be triggered by a dysbiotic gut microbiota in a mouse model (Schaubeck et al., 2015).

Altogether, these observations strongly argue for a causal effect of LOMD in several Human conditions.

### The western lifestyle may explain the reduced diversity of the intestinal microbiota

LOMD is a feature of industrialized countries. Among the many candidate risk factors causing the LOMD, some of them can be highlighted, such as lifestyle, eating behaviors, disruption of biological clock and antibiotic consumption (Figure 1).

The microbiota of Malawian and Venezuelan people are more diversified than their US children and adult counterparts (Yatsunenko et al., 2012). Similar differences were found when comparing Bangladeshi to American children (Lin et al., 2013). More recently, the analysis of gut microbiota patterns of rural Papua New Guineans compared with those of people from USA showed that westernization may decrease bacterial dispersal rates and alter the microbiota structure (Martínez et al., 2015). The Human hunter-gatherers Hadza of Tanzania—where people still live outside without access to antibiotics and treated water—had higher levels of microbial richness and biodiversity than Italian urban controls (Schnorr et al., 2014).

Different eating behaviors that are known to rapidly affect the intestinal microbiome (David et al., 2013) are obvious possible explanations. For example, fiber consumption may explain the differences observed between children from Burkina Faso and Italy (De Filippo et al., 2010). Recently, the impact of diet on the microbiota has been reviewed, and it turns out that fiber rich diet enhances gut microbiota diversity (Simpson and Campbell, 2015; for a more complete critical review on gut microbiota and diet, see Graf et al., 2015). However, these differences might not only be due to diet (Wu et al., 2016) and alternative hypotheses may be raised. For example, the practice of cesarean section has grown steadily since the second half of the twentieth century in Western countries, reaching an average of 26.9% of birth in OECD countries (OECD, 2013) but only 5% in developing countries (Betrán et al., 2007). Children born by cesarean section have a LOMD when compared to those born vaginally (Jakobsson et al., 2014).

Modern lifestyle might trigger a disruption of the biological clock with consequences for the host and the microbiota. Several characteristics of modern life style such as working shifts, stress, jet lag, unusual feeding patterns have been shown to disrupt the biological clock. The link between microbiota, its host and diurnal oscillation was well-described for the squid-*Vibrio fischerii* symbiosis (Wier et al., 2010). More recently, the intestinal microbiota in both mice and humans has been shown to exhibit diurnal oscillations, that when disrupted, led to a LOMD and dysbiosis (Thaiss et al., 2014).

Antibiotics (ATB) are other candidate risk factors. Naturally produced by many microorganisms, ATB have been developed by humans after the 30's. Beyond human medicine, antibiotics are now widely used especially for animal rearing, to the point where they are now markers of human economic activity. Humans cause a massive spread of ATB in the environment with significant impacts on the bacterial ecosystems of water, soil, and plants (Sarmah et al., 2006; Dolliver and Gupta, 2008; Martinez, 2009). ATB profoundly alter the structure of the intestinal microbiota (Buffie et al., 2012) and reduce the bacterial diversity (Dethlefsen et al., 2008; Pérez-Cobas et al., 2013; Zhao et al., 2014). Of note, ATB use during infancy and childhood is associated with increased incidences of asthma, atopic dermatitis, MS, IBD, juvenile idiopathic arthritis and obesity (Zeissig and Blumberg, 2014; Horton et al., 2015; Schwartz et al., 2015).

## LOMD is a hallmark of a defective equilibrium between predators and their preys

### What shapes microbial communities?

Whatever the relevant environmental risk factor(s) playing a role in industrialized countries, the way it impacts the composition of the gut microbiota remains to be explained.

**Microbial communities** harbor a wide range of ecological interactions which may be of five different types including (i) mutualism, where both the participants are benefited; (ii) amensalism, where one organism is inhibited or destroyed and the other is unaffected; (iii) commensalism, where one partner gets the advantage without any help or harm to the other; (iv) competition, where both the participants harm each other; and (v) predation and parasitism, where one gets benefited out of the other (Faust and Raes, 2012). All of these interactions are supposed to shape the community assembly. For instance, cooperation through metabolic exchange between species has been shown to be predictive for some co-occurrences (Zelezniak et al., 2015). However, the way diversity is shaped and persists is still poorly understood for the complex network of interactions within the gut.

Thank to phylogenetic approaches, knowledge on mechanisms governing microbial communities has improved over the last few years, filling the gap between knowledge on macrobial and microbial ecosystems. According to Vellend's conceptual synthesis of community ecology (Vellend, 2010), diversity at local scales (**alpha diversity**) is shaped by a combination of four processes: dispersal, local diversification, environmental selection and ecological drift (or demographic stochasticity; Costello et al., 2012). Inside the gut ecosystem, such processes could drive the microbiota community and specifically the microbiota diversity toward short-term and long-term co-evolution. Then, we thought to be inspired by macro ecological theories to encompass to what extend predation could play a role in driving gut microbiota diversity.

### The human activities impact the predator/prey equilibrium

In macro ecology, it is now recognized that human activity causes a decrease in biodiversity of unprecedented proportions (Butchart et al., 2010). Among the many parameters that are under the pressure from Human activities, a major one is the loss of large predators, i.e., those who are at the top of the food chain in a given ecosystem. In 1966, Paine was the first to observe a decrease of mussel species in a marine ecosystem when removing the starfishes (Paine, 1966). He hypothesized that “the local biodiversity was directly related to the efficiency with which predators prevent the monopolization of the major environmental requisites by one species.” Similarly, sea otters protect kelp forests from damage by sea urchins (Szpak et al., 2013). Australian natives introduced dingo (*Canis lupus Dingo*) at least 3500 years ago. As it preyed upon livestock, the dingo has been the target of extermination programs for the last two centuries. Hereby, it was seen that the loss of dingoes has resulted in the depletion of plant biomass through a dysregulation of herbivore population (Letnic et al., 2012). Indeed, the impact on ecosystems of the decline of the seven largest carnivores in the world has been recorded in recent decades and the disappearance of a large carnivore always alters deeply the ecosystem and more particularly, it reduces its biodiversity (Ripple et al., 2014).

### Predators and preys are in a dynamic equilibrium in healthy ecosystems

**Figure 2:**
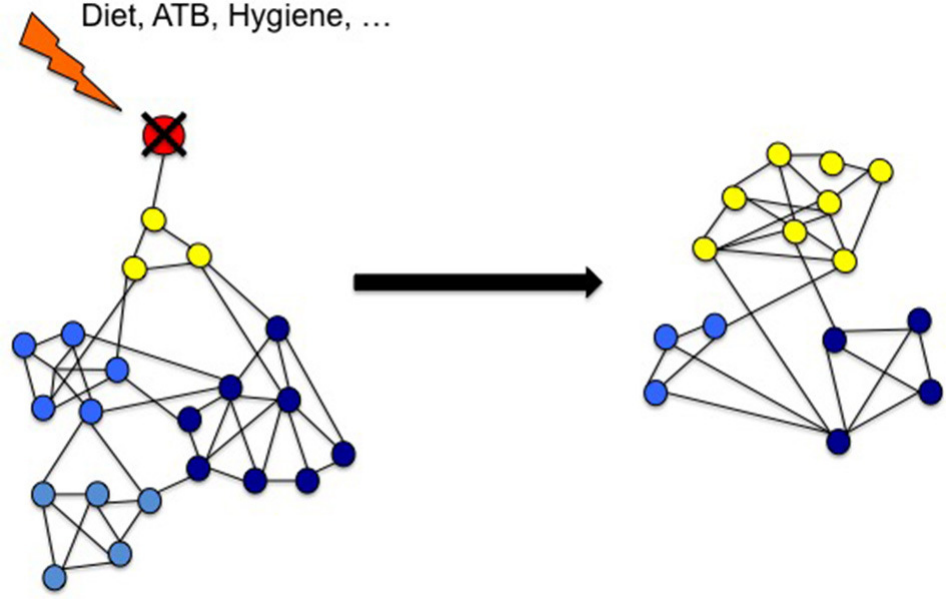
**Environmental factors reduce microbial diversity because of the loss of predatory species**. Gut microbiota may be seen as a complex network of many interacting species (nodes) with several kinds of interactions (links). Predators (in red) are key species that maintain the diversity of the microbiota by direct impact on preys (yellow) and indirect effect on other related species (blue). According to Voltera equations, loss of predators causes an increase number of preys but a loss of diversity. We propose that in industrialized countries environmental risk factors reduces the predators in the network causing LOMD.

It thus appears that experimentally, species diversity can be maintained by predation. In the absence of predators, a few dominant species can grow rapidly and then supplant many of the other species, limiting the amount of their available resources. Conversely, the presence of predators limits the population of dominant species. The current working paradigm for community dynamics -understudied in the human gut- is the Lotka Voltera model. Colloquially known as Kill-the-Winner, the Lotka Voltera model predicts that predators will rapidly and drastically reduce the population of the most abundant species, preventing the best competitors from building up a high biomass. The role of a predator population in maintaining species diversity has been assessed by computer simulations, which displayed the robustness of the “Lotka-Volterra-like” cycle and implies that frequent predation might be a mechanism to maintain species diversity in nature. Moreover, combining the classical Lotka–Volterra model of population dynamics with regression techniques provides a mechanistic scheme that can be used to construct predictive models of ecosystem dynamics. Such an approach has been successfully applied to the gut microbiota ecosystem to show which network elements are disrupted during ATB course and predict to what extend Clostridium difficile infection (CDI) can arise (Stein et al., 2013). In the same way, it should be possible to design a mathematical models including predation within the gut microbial community (Figure 2). Such model would be validated by new *in vitro* data issued from studies on microbial predation in animal's gut.

### Lack of resilience and stability are major consequences of LOMD on ecosystems

The **stability** of microbial ecosystem can be analyzed through predictive or mechanistic models. On one hand, microbial communities tend to be more phylogenetically clustered than expected by chance. For instance, in aquatic ecosystems, the response to disturbance has been linked to the community network (Hunt and Ward, 2015). Then, a predictive model can be drawn to predict reactions to disturbance in a particular community. Within the gut microbiota community, where microbes compete and cooperate, such network has also been highlighted. Indeed, co-occurrence of microbial species are commonly measured in the gut microbiota (Faust et al., 2012).

**Figure 3:**
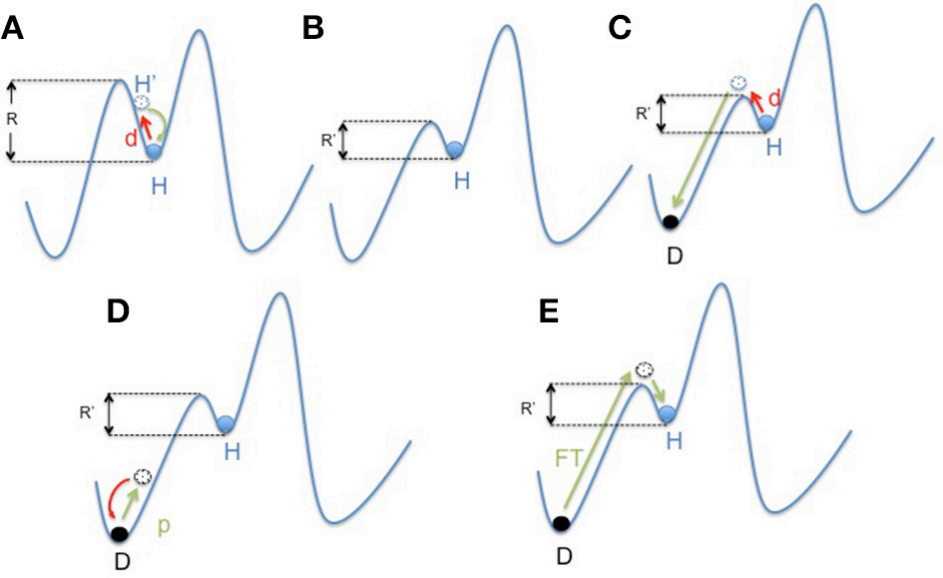
**Mapping concept of microbiota dysbiosis and resilience**. **(A)** In a healthy state, the gut microbiota ecosystem (represented by a ball) is in a steady state (H). The depth of the well in which the ball is located represents its resilience (R). In case of disturbance (d; i.e., ATB course or digestive infection), the gut microbiota changes (H′) and then get back to its anterior state (H). **(B)** Environmental factors (i.e., western lifestyle) negatively impact the diversity of the microbiota resulting in a decrease of resilience (R′ < R). However, the microbiota still remains in an apparent healthy state, due to the functional redundancy of the ecosystem (H). **(C)** But in this new situation, the same disturbance (d) results in a shift of the gut microbiota balance to an other state called dysbiosis **(D)**. This new state is also a steady state, but it impacts negatively the individual condition. It represents an “unhealthy” state. **(D,E)** “Rebiosis” with respective effect of probiotics administration and fecal transplantation on the the gut microbiota ecosystem. A single strain (probiotic) fails to restore a “healthy” ecosystem (**D**), but a radical change of the ecosystem through fecal transplantation could be able to achieve this goal (**E**). p, probiotics; FT, Fecal Transplant.

On the other hand, in large ecosystems, a decrease of **resilience** is often associated with the lack of biodiversity. Resilience is defined as the amount of disturbance a system can absorb while remaining in the same functional state. It also refers to the ability of an altered ecosystem to reorganize and renew in case of failure. Through the diversity/versatility of each species response to external or internal changes, a high biodiversity helps to maintain a stable ecosystem function called “robustness” or **stability** (Elmqvist et al., 2003). Coral reefs around Jamaica are an example of such a phenomenon (Hughes, 1994). Overfishing led to a loss of diversity in the functional group of “grazers,” but that loss was made up by an increase in sea urchins, which offsets the loss of other herbivores and maintained corals as in “healthy state.” However, when a pathogen reduced the population of sea urchins (Lessios et al., 1984; Nyström et al., 2000) there were no grazing species left to prevent the invasion of algae that leads to the “undesirable state.” In other words, the “diversity of response increases the tolerance for management of mistakes” (Elmqvist et al., 2003; Figures 3A–C).

## Predators inside the gut

Similarly to macro-ecosystems where the influence of human activity reduces biodiversity by the reduction of large predators, we hypothesize that the Western lifestyle reduces the diversity of the intestinal microbiota by loss of bacterial predation. Several microorganisms such as protists, bacteriophages and predatory bacteria are known to be predators of components of the gut microbiota.

### Protists

Amoebas have developed complex interactions with bacteria during evolution (Cosson and Soldati, 2008). Basically, their interaction is a predator-prey model for which several parameters have been described. Differences in susceptibility to predation differentiate bacterial species (e.g., medium-sized bacteria are most vulnerable to predation while bacteria with small and large filamentous forms are partially resistant to predation). Direct and indirect influences of predation on the conditions of bacterial growth (substrate supply) or bacterial competition (elimination of competitors) are also important. In soils, this relationship seems even more complex, since under favorable environmental conditions, the interaction between amoebae and non-virulent bacteria causes lysis of the bacteria, while the interaction with low-virulent bacteria causes a symbiotic relationship or amoebic lysis (Ronn et al., 2002). Interestingly, the hypothesis that protist predation could shape the gut microbiota diversity has been recently supported by the discovery of an association between the absence of commensal amoebas in the intestine of West African subjects and LOMD (Morton et al., 2015).

### Bacteriophages

Bacteriophages, or phages, are the most abundant biological group on Earth. Phages essentially belong to two categories, virulent and temperate. The virulent phage lifecycle consists in replicating in, and then lysing its bacterial host. Temperate phages can alternate between two lifestyles: they either lyse their host, like virulent phages, or establish a symbiosis with it, the so-called “lysogeny state.” In most ecosystems, where phages outnumber bacteria by a 10-fold factor, predation by phages allows maintaining bacterial diversity (Rodriguez-Valera et al., 2009). However, in humans, only 10^8^ phages vs. 10^12^ bacteria /g of feces were reported with a preferentially lysogenic phenotype in the colon of healthy people (Mills et al., 2013). Moreover, phages cannot move to go and meet their prey and are therefore subject to a “random collision.” Consequently, viral-bacterial predator dynamic, evident in a number of other characterized ecosystems as aquatic environment (Rodriguez-Valera et al., 2009), could be absent in the distal intestine of healthy individuals (Reyes et al., 2010, 2013). Thus, they may not be considered “key predatory.”

### Predatory bacteria

To date, the most studied predatory bacteria belong to the Bdellovibrio-and-like organisms group called BALOs. The first report of *B. bacteriovorus*, gen. et sp. n. was made by Stolp and Starr in 1963 (Stolp and Starr, 1963) as a predatory and bacteriolytic microorganism. *Bdellovibrio bacteriovorus* (Bdello) is a microaerophilic species from the δ Proteobacteria group naturally present in soils (rhizosphere) and freshwater but also in the human intestine (Iebba et al., 2013). It is a highly motile bacterium that seeks out a prey bacterium and invades it. The ensuing intracellular growth and replication lead to the lysis of the prey bacterium and then motile predators are released (Strauch et al., 2007). It is a non-specific predator of Gram-negative bacteria but it is not pathogenic for higher organisms (Dashiff et al., 2011). Beyond this direct predation against gram-negative bacteria, it even impacts the growth of Gram positive bacteria such as *Staphylococcus aureus* (Iebba et al., 2014; Monnappa et al., 2014).

Despite its broad spectrum of activity on many gram negative bacteria (Dashiff et al., 2011; Kadouri et al., 2013), its impact on the intestinal microbiota has not been studied. So far, it has mainly been studied as a potential alternative to antibiotics (Sockett and Lambert, 2004; Dwidar et al., 2012) for ocular (Shanks et al., 2013), periodontal (Van Essche et al., 2011), or respiratory infections (Iebba et al., 2014). The only report of oral administration of Bdello showed a 10-fold decrease of *Salmonella enteritidis* and a reduction of morphological abnormalities associated with the cecum infection in infected chicken (Atterbury et al., 2011). The effects of Bdello on oral microbiota were recently analyzed using high throughput-sequencing methods (Loozen et al., 2015). *Ex vivo* experiments performed on sub-gingival plaque and saliva samples from periodontitis patients demonstrated changes in the overall ecology. Unfortunately, the effect on bacterial diversity was not specifically assessed. However, the evidence of coevolution of Bdello and its preys according to the Red Queen theory has been made by a long-term experiment involving Bdello and its prey *Pseudomonas fluorescens* (Gallet et al., 2009), indicating that Bdello could drive the biodiversity in the ecosystem when present.

## Predators, microbiota diversity and health: Clinical perspectives

### Microbiota diversity and the host immune system: A key point

Predatory-preys interactions were operating among bacteria long before the appearance of animals on earth. Then, animals provided new surroundings for bacteria, resulting in a more complex interaction between host cells and microorganisms. For instance, gut microbiota in vertebrates' gut adapts to the host diet over daily time scales, as we already mentioned (Graf et al., 2015), but even over evolutionary scales. This allowed animals to face environmental changes as they were able to digest a broad variety of biomolecules (Ley et al., 2008). Moreover, it has been proposed that this microbial functional diversity would have been beneficial for the host in an evolutionary perspective since it might have shaped the adaptive immune system (McFall-Ngai, 2007; McFall-Ngai et al., 2013).

Although many studies assessed the impact of single bacterial species on inflammatory markers, few of them looked at the relationship between microbiota diversity and its consequences on the immune system. One of the major components of the adaptive immune system are the CD4+ regulatory T (Treg) cells, which express the transcription factor Foxp3 and play an essential role in maintaining the immune homeostasis. Gnotobiotic mice colonized with 3 strains of *Clostridium* were shown to display a profile of Treg induction intermediate between axenic animals and mice inoculated with 46 strains. This observation suggests that an optimal induction of Tregs requires a set of metabolites that are more efficiently produced by a panel of *Clostridium* strains (Atarashi et al., 2011). On the other hand, axenic mice and those with low microbiota diversity develop high levels of serum IgE early in life (Cahenzli et al., 2013). Finally, an antibiotic treatment with vancomycin reduced the microbial diversity and increased the severity of asthmatic disease in mice (Russell et al., 2012).

### Crohn's disease: A typical example of the interaction between western lifestyle and dysbiosis, where Bdello could play a role

CD typically represents a chronic inflammatory disease whose incidence increased sharply in Western countries (Molodecky et al., 2012) with large variations between Westernized and developing countries (Ng et al., 2013). It is also a disease for which epidemiological studies have linked low exposure to infectious agents and disease occurrence. For example, the farm life in childhood would protect against the disease (Amre et al., 2006). ATB taken in childhood increase the risk of CD (Shaw et al., 2010; Hviid et al., 2011; Ungaro et al., 2014). CD is also associated with a dysbiosis with a significant reduction in bacterial diversity (Manichanh et al., 2006; Dey et al., 2013; Sha et al., 2013). Finally, the presence of Bdello in stools and intestinal biopsies of children with CD has recently been studied. Only one out of nine patients carried Bdello while healthy eight controls were all positive (Iebba et al., 2013).

What would be the consequences of Bdello decrease in CD? Firstly, Bdello having a predation predominant on Gram negative bacteria, its absence could result in a proliferation of “pathobiont” bacteria, like Adherent and Invasive *Escherichia coli* (AIEC) or Yersinia species which are suspected to play a role in CD lesions (Carrière, 2014). Secondly, it can explain the observed increased proportion of Gram-negative symbionts (for example *Bacteroides* from the Bacteroidetes phylum) with a subsequent decrease of Gram positive bacteria such as *Faecalibacterium prausnitzii* from the *Clostridium leptum* group within the Firmicutes observed in CD patients. As a whole, CD could thus represent a prototypic example of the proposed theory.

### “Rebiosis” or how to restore the microbial diversity?

Since the discovery of disease-associated dysbiosis, it is now accepted that a therapeutic goal is to restore a “healthy” or balanced microbiota, notably by improving the gut microbiota diversity. Several therapeutic attempts have been considered. Elie Metchnikoff first wrote about the health benefits of probiotics. He observed that Bulgarian peasants who consumed large amounts of fermented milk, the early form of yogurt, lived longer and healthier. For over a century, probiotics have been used in a large number of diseases, but with disappointing results (Quigley, 2010), even in CD (Orel, 2014). The rationale could be that providing one or a handful of strains may not be sufficient to restore a functionally compromised ecosystem (Figure 3D). As a matter of fact, it was recently shown that some probiotic strains delivered to the mothers failed to increase the microbiota diversity in their offspring's (Dotterud et al., 2015).

To restore the diversity, transplantation of fecal microbiota has been proposed. This drastic therapy, described in China as early as in the fourth century, was occasionally used as a treatment for severe and ATB resistant *Clostridium difficile* colitis during the twentieth century (Vrieze et al., 2013). Recently, the European Society of Clinical Microbiology and Infectious Diseases has included this approach for the treatment of CDI (Debast et al., 2014) (Figure 3E). In the same line, the “Repopulate” study proposed a cocktail of 33 species cultured from stools of a single donor. Two patients were effectively treated for CDI and the bacterial populations persisted in the recipient colon for over 6 months after the transplantation.

Alternatively, If our theory is true, an option could be to reintroduce predators in order to restore the microbial diversity and health.

## Conclusion

Over the last few years, data linking LOMD and Human diseases associated with the westernized lifestyle has been accumulating. We hypothesize that the modern western lifestyle may be associated with a loss of predators within the microbiota ecosystem as demonstrated in macro-ecological models. According to this hypothesis, the lack of predation and LOMD could lead to an unstable microbial ecosystem. The consequent instability could favor the emergence of dysbiotic microbiota giving rise to immunological or metabolic diseases. If demonstrated, this point of view might bring a significant impact on understanding and treating these diseases.

## Author contributions

AM, Hypothesis conception and drafting manuscript; ML, Critically revising the manuscript; JH, Critically revising the manuscript.

## Funding

We acknowledge the financial support of the “Investissements d'Avenir programme ANR-11-IDEX-0005-02, Sorbonne Paris Cite,” Laboratoire d'excellence INFLAMEX, Inserm and Assistance Publique des Hôpitaux de Paris.

### Conflict of interest statement

The authors declare that the research was conducted in the absence of any commercial or financial relationships that could be construed as a potential conflict of interest.

## Glossary

- **Intestinal dysbiosis:** Distinct definitions of dysbiosis can be found from the literature. In practice, dysbiosis refers to microbiota that differs from the one of healthy people. We chose the definition of Petersen and Round (Petersen and Round, 2014) because it seems relevant here since it is linked to its impact on host immunity. According to, dysbiosis can be defined by three main features, (i) a loss of beneficial microbial organisms, (ii) the expansion of pathobionts, and (iii) a loss of microbial diversity.

- **Diversity metrics:**

- - **Richness:** Number of taxonomic units (e.g., species) in a community, standardized to the number of individuals sampled.
- - **Alpha diversity:** An inventory metric that expresses the amount of local diversity or diversity at the smallest spatial scale of analysis (e.g., Intra individual gut microbiota diversity). Assessed by Shannon or Simplon index for example. LOMD refers to alpha diversity.
- - **Beta diversity:** A differentiation diversity metric that refers to turnover at the landscape scale, i.e., turnover between local populations (e.g., inter-individual gut microbiota diversity within the same population): not used in this paper.
- - **Gamma diversity:** is a similar metric used to refer to diversity at the regional scale (e.g., between different population: westernized vs. non westernized): not used in this paper).

- **Community:** A group of potentially interacting species that co-occur in space and time (e.g., gut microbiota).

-**Resilience and stability:** The stability of an ecosystem is defined as a community's response to disturbance. It depends on the resistance (insensitivity to disturbance) and the resilience of the ecosystem (Shade et al., 2012). The resilience is the rate at which a microbial community returns to its original composition after being disturbed (Allison and Martiny, 2008). Then, it represents a measure of the ability of this system to absorb changes and still persist. In this definition, resilience is the property of the system and persistence or probability of extinction is the result (Holling, 1973).
